# Varian ethos online adaptive radiotherapy for prostate cancer: Early results of contouring accuracy, treatment plan quality, and treatment time

**DOI:** 10.1002/acm2.13479

**Published:** 2021-11-29

**Authors:** Mikel Byrne, Ben Archibald‐Heeren, Yunfei Hu, Amy Teh, Rhea Beserminji, Emma Cai, Guilin Liu, Angela Yates, James Rijken, Nick Collett, Trent Aland

**Affiliations:** ^1^ Icon Cancer Centre Wahroonga Sydney Adventist Hospital Wahroonga New South Wales Australia; ^2^ School of Information and Physical Sciences University of Newcastle Newcastle New South Wales Australia; ^3^ Icon Cancer Centre Gosford Gosford New South Wales Australia; ^4^ Icon Cancer Centre Windsor Gardens Windsor Gardens South Australia Australia; ^5^ Icon Core Office South Brisbane Queensland Australia

**Keywords:** adaptive therapy, deformable registration, machine learning

## Abstract

The Varian Ethos system allows for online adaptive treatments through the utilization of artificial intelligence (AI) and deformable image registration which automates large parts of the anatomical contouring and plan optimization process. In this study, treatments of intact prostate and prostate bed, with and without nodes, were simulated for 182 online adaptive fractions, and then a further 184 clinical fractions were delivered on the Ethos system. Frequency and magnitude of contour edits were recorded, as well as a range of plan quality metrics. From the fractions analyzed, 11% of AI generated contours, known as influencer contours, required no change, and 81% required minor edits in any given fraction. The frequency of target and noninfluencer organs at risk (OAR) contour editing varied substantially between different targets and noninfluencer OARs, although across all targets 72% of cases required no edits. The adaptive plan was the preference in 95% of fractions. The adaptive plan met more goals than the scheduled plan in 78% of fractions, while in 15% of fractions the number of goals met was the same. The online adaptive recontouring and replanning process was carried out in 19 min on average. Significant improvements in dosimetry are possible with the Ethos online adaptive system in prostate radiotherapy.

## 1 INTRODUCTION

In online adaptive radiotherapy (OART), the treatment plan is adjusted to the specific anatomy on a given day to ensure the optimal trade‐off between irradiating the treatment target and sparing of normal tissue. OART has the potential to result in significant clinical benefits for prostate patients.^1^, ^2^, ^3^, ^4^, ^5^, ^6^, ^7^, ^8^, ^9^ A study by Ahunbay et al.^1^ reported a 13% increase in minimum PTV dose and a 13% decrease in equivalent uniform dose to the rectum when using different online adaptive strategies for prostate.

The technical challenges of utilizing a full daily replan for OART are significant.^10^ An image of the daily anatomy needs to be acquired, the anatomy contoured, and a plan generated based on the anatomy of the day. The plan then needs to be evaluated by clinical staff and subject to a quality assurance (QA) process, before being delivered to the patient. This all needs to be performed in a sufficiently short timeframe such that the anatomy being adapted to does not change from the initial image. This timeframe depends on the anatomy being treated; but in the pelvis, significant changes in bladder size can occur within 15 min.^11^, ^12^

Until recently, the technical and logistical challenges of OART made it practically infeasible for most radiotherapy centers. The introduction of artificial intelligence (AI)^13^, ^14^ and graphics processing unit (GPU) based calculation engines^15^, ^16^, ^17^ have allowed for the many steps in an online adaptive workflow to be performed in the timeframe required for OART. The Varian Ethos system (Varian Medical Systems, Palo Alto, CA) was recently developed as a completely self‐contained online adaptive solution, and has been reported to be capable of performing adaptive treatments within 15–20 min.^18^, ^19^, ^20^ The dosimetric accuracy of the Ethos treatment planning system has previously been comprehensively verified.^21^

The workflow used on Ethos for online adaptive prostate patients involves the use of “influencer” structures that are initially auto‐contoured using AI. The influencer structures are then adjusted by the user if necessary and used to create a structure‐guided deformable image registration (DIR) between the planning computed tomography (CT) scan and the acquired cone beam computed tomography (CBCT) scan. An elastic DIR is also created between the planning CT and CBCT. The gross tumor volumes (GTVs) and clinical target volumes (CTVs) are propagated from the planning CT to the CBCT using the structure‐guided DIR if the GTV/CTV is considered mobile, or the elastic DIR if considered nonmobile.^20^, ^22^ The elastic DIR is also used to both propagate noninfluencer organs at risk (OARs) and generate a synthetic CT by deforming the planning CT into the CBCT geometry. This synthetic CT uses the planning CT Hounsfield units (HU) to provide the density information for dose calculations performed in the treatment geometry, and its accuracy is validated on a patient‐specific basis by visually checking structure agreement with the CBCT. A plan is generated based upon a predefined “planning directive” optimized to the anatomy of the day and referred to as the adaptive plan. The original treatment plan (reference plan) has an automated match applied, then is recalculated based on the anatomy of the day and referred to as the scheduled plan. The user selects either the scheduled or adaptive plan for the treatment. The plan selected receives pretreatment calculation‐based QA, and posttreatment delivery log file‐based QA using Mobius (Varian Medical Systems, Palo Alto, CA), an established patient‐specific QA solution.^23^ A verification image can be acquired after completing the adaptive process and before treatment, to account for any intrafraction motion, and the treatment is then delivered to the patient.^24^

Direct validation of many of the steps in the Ethos adaptive process is difficult because they are not able to be performed in isolation, nor are the structure guided and elastic DIRs able to be exported or visualized. The system is generally designed as a “black box,” where only the inputs and outputs are available for interrogation. For this reason, other early studies^18^, ^19^ have taken the form of analyzing DIR and AI outputs clinically.

Due to the novelty of the Ethos system, there is little published research investigating an optimal method or expected results for prostate OART using Ethos. Yoon et al.^19^ performed an initial evaluation of the Ethos system on retrospective head and neck patient data, finding 82% of contours were subjectively scored as ≥4 out of 5, where 1 represented unacceptable contours, and 5 represented perfect contouring. A recent study by Sibolt et al.^18^ presented preliminary data on the clinical implementation of the Ethos system across a range of pelvic sites. Eight retrospective prostate plans were included, and every fifth fraction was analyzed. They found that either no or minor edits to influencer contours were required in 76% of fractions, and the adaptive plan was selected in 88% of fractions. However, the number of fractions analyzed in this study was small, nodal information was not presented separately, and prostate bed data were excluded.

The aim of this study is to report early results on the accuracy of automated contouring, plan quality, and treatment fraction timing for Ethos OART to the prostate. A range of clinical metrics for each fraction are reported for intact prostate, prostate and nodes, and prostate bed and nodes treatments from one institution. This will assist centers to gain an understanding of the dosimetric benefits possible with this treatment technique, as well as a starting point when developing their own OART workflow with this new technology.

## 2 METHODS

### 2.1 Study dataset

Eighteen patients were selected for the study dataset. This was made up of 12 patients previously treated on a Halcyon that had a simulated treatment performed on the Ethos treatment emulator, a nonclinical version of the Ethos software setup for treatment fraction simulations. This retrospective dataset was supplemented with six clinical adaptive cases treated on the Ethos system.

At our institution, prescribed doses and organ at risk (OAR) limits are primarily based on the eviQ guidelines (an Australian evidence‐based and peer‐reviewed guideline).^25^ Contouring was adjusted and plan selection carried out based on standard plan criteria. The 12 retrospective patients consisted of four intact prostate cases (prescribed 60 Gy/20 Fx), four prostate bed and node cases (prescribed 66 Gy/33 Fx), and four prostate and node cases (prescribed 78 Gy/39 Fx). Within the retrospective dataset, every second fraction was simulated in the treatment emulator, thus analyzing images over the entire treatment course. The total retrospective dataset consisted of 182 simulated fractions.

The six clinical adaptive cases included two intact prostate cases, two prostate and node cases, and two prostate bed and node cases. These patients were treated using the same online adaptive workflow tested on the retrospective patients. This dataset included every fraction, 184 in total.

Considering the extended treatment times on Ethos, the patient comfort was ensured by marginally reducing pretreatment bladder filling from 500 ml (used previously within the institution) to 400 ml. Note that the retrospective dataset used the previous 500 ml filling, while the clinical patients used the new 400 ml filling. The full test dataset is summarized in Table 1.

**TABLE 1:** Summary of dataset used for study

**Treatment site**	**Number of retrospective patients**	**Number of clinical patients**	**Total fractions**	**Number of patients with implanted fiducials**	**Comments**
Intact prostate	4	2	84	3	2 with hydrogel spacer
Intact prostate and nodes	4	2	150	3	1 with hydrogel spacer
Prostate bed and nodes	4	2	132	0	Surgical clips visible in 3 cases, 1 patient with prosthetic hip

### 2.2 Reference plan generation

To generate a plan in Ethos, a set of clinical goals is required. The goals have a dual function of being the clinical intent of the plan, as well as the goals used in the optimization. Each goal has a minimum acceptable value and an ideal value. Generally, the clinical goal (based on eviQ^25^) was entered as the minimum acceptable value, and an ideal value (somewhat equivalent to an optimization goal) was entered as the ideal value. More information regarding the Intelligent Optimization Engine (IOE) is provided by Archambault et al.^20^ All planning directives used in the study had at least three minimum dose goals per prescribed dose level (CTV D98, PTV D98, and D95).

All plans used rectum and bladder as influencer structures, and cases with an intact prostate also used the prostate and seminal vesicles as influencer structures. CTVs were determined by the radiation oncologist based on CT images. For intact prostate patients, CTVs were created as independent structures, not derived from the prostate and seminal vesicle structures. All other structures were determined by an RT and reviewed by the radiation oncologist. As this was a first step in developing OART for prostate at our institution, standard IGRT margins based on eviQ guidelines^25^ were used, without any reductions.

A 7, 9, or 12 field IMRT plan was generated for each case. The study included plans from both Ethos v1.0 and v1.0 MR1. VMAT was not used as it has been observed to give inferior plan quality in the current version of Ethos.^18^

### 2.3 Treatment

All staff involved in performing treatments for this study (either retrospective or clinical) underwent vendor supplied Ethos training in addition to in‐house credentialling. The in‐house credentialling included graded assessments of workflow knowledge, delineation of pelvic anatomy on CT images, and Ethos adaptive treatments on the emulator.

Retrospective emulator treatments were carried out by a physicist or radiation therapist. Users were instructed to match the influencers with the anatomy seen on the CBCT, while targets and noninfluencer OARs were assessed and adjusted if changes were expected to make a clinical impact to the plan. In practice, this meant changes to targets and noninfluencer OARs smaller than 2 mm were not applied, and changes to noninfluencer OARs further than 3 cm from the PTV were not applied.

Clinical treatments were carried out by a team consisting of at least two radiation therapists and a physicist under the supervision of the treating radiation oncologist. Depending on progress through the treatment course, the radiation oncologist was available in‐person or online. A postadaptive pretreatment verification CBCT scan was acquired between completion of the adaptive planning process and treatment delivery, with assessment of intrafraction motion during the adaptive planning timeframe. If deemed necessary by the clinical team, translational couch shifts were applied before delivering the treatment.

### 2.4 Metrics assessed

The metrics assessed for each delivered fraction are outlined below, they capture the frequency and magnitude of contour edits, changes in plan quality, and time required for OART to the prostate.

#### 2.4.1 Contour accuracy

The frequency and magnitude of edits for influencers, targets, and noninfluencer OARs were recorded as an indicator of amount of manual intervention required. They are based on the method applied by Sibolt et al.^18^ and act as a surrogate for automated contouring accuracy. For each structure in each fraction, users were required to categorize the editing required as either:

1. No edits required—no changes made to the structure.
2. Minor edits required—less than 10% of slices requiring small edits.
3. Moderate edits required— > 10% of slices requiring minor edits, or major edits required to a small number (10%) of slices.
4. Major edits required—edits not described in above categories, up to and including deletion of structure and recontouring.
5. Not applicable—not relevant to the fraction or not assessed.

#### 2.4.2 Plan quality

A range of plan quality metrics were analyzed for each fraction. To assess average plan quality for the adaptive plan as compared to the scheduled plan, the number of clinical goals met at both the minimum acceptable level and the ideal level was recorded for each fraction. The value for the scheduled plan was then subtracted from the value for the adapted plan, giving the difference in the number of goals met, where a positive value indicates that more goals were met for the adaptive plan, and a negative value indicates that more goals were met for the scheduled plan. This metric was chosen because: it is calculated from the clinical goals which are indicative of clinical outcomes, it combines all plan metrics into a single comparative value for each fraction, and it allows specific goals used to vary between treatment sites. Note that the use of this metric means that all clinical goals are considered equal, whereas in clinical practice, the oncologist will usually prioritize some metrics over others. The frequency of adaptive plan selection was also recorded for each fraction. To investigate how the goals differed between the adaptive and scheduled plans, the median PTV and OAR goals were analyzed over the treatment course for a representative patient. Statistical significance was determined using a Wilcoxon signed rank test and the pseudo‐median (Hodges–Lehmann) displayed, as some DVH parameters were not normally distributed. The null hypothesis (*H*_0_) was that there was no difference between the adapted and scheduled plans, with significance set at *p* = 0.05.

#### 2.4.3 Adaptive time

The time for the retrospective emulator treatments was recorded from simulated completion of image acquisition to plan acceptance. Clinical treatment time was recorded from the time of opening the patient on the Ethos treatment machine to the time of closing the patient. In a small number of clinical treatments, the patient was given additional time or taken off the couch to release rectal gas; these fractions were excluded from the timing dataset.

## 3 RESULTS

### 3.1 Influencer contouring accuracy

**FIGURE 1:**
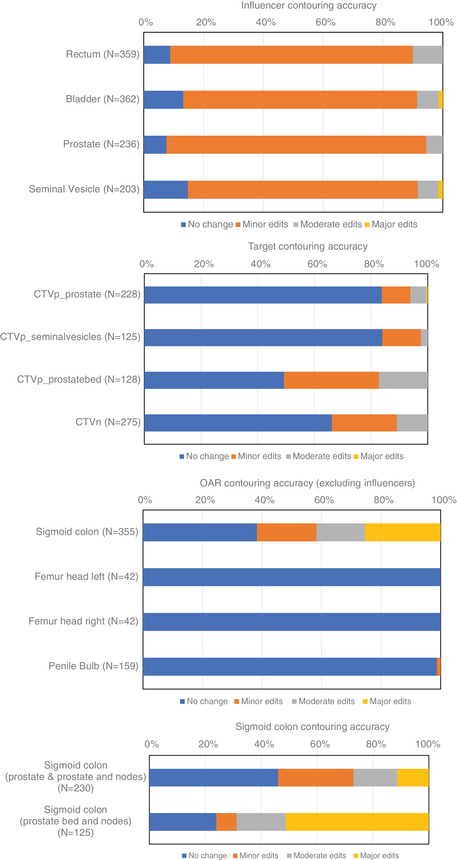
Frequency of edits required for (a) influencer structures; (b) target structures; (c) noninfluencer OAR structures; and (d) frequency of sigmoid colon editing by treatment site

The frequency and magnitude of edits are shown for each influencer in Figure 1a. No edits were required in 11% of fractions overall, and minor edits were required in 81% of fractions overall.

The bladder contouring of the patient with a hip prosthesis was significantly worse and accounted for all the fractions, where the user had to make major edits to the bladder contour, as well as a large number of fractions with moderate edits. The bowel influencer had previously been tested and was found to give inconsistent results. It was not used in the planning intents in this study.

### 3.2 Target contouring accuracy

Figure 1b shows the frequency of CTV editing required. As can be seen for a prostate target, no change is required more than 80% of the time. For cases involving nodes and prostate bed, the frequency of CTV editing increases significantly. Overall the percentage of CTVs requiring no change was 72%, and requiring no or minor changes was 91%.

### 3.3 OAR contouring accuracy (excluding influencers)

The frequency of editing the noninfluencer OARs is shown in Figure 1c. Noninfluencer OARs were assessed in every fraction they were available for assessment, however depending on the priority assigned to the structure in planning, in many cases they were not available for assessment. The sigmoid colon contouring required changes much more frequently than any other structure. No comparable data for noninfluencer OARs have been reported in the literature.

By separating the sigmoid colon contouring data by treatment site, treatment site‐specific differences can be visualized. It was found that there were considerable differences in sigmoid colon contouring accuracy for the prostate bed cases, shown in Figure 1d.

### 3.4 Differences in number of clinical goals met

**FIGURE 2:**
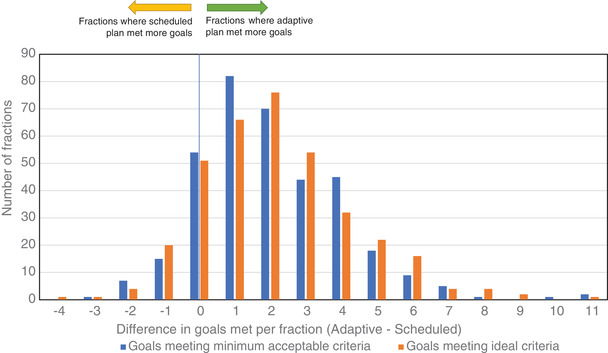
Histogram of differences in number of planning clinical goals met per fraction

A histogram of the differences in the number of goals met over all fractions analyzed is shown in Figure 2. The distribution is strongly positively skewed, indicating that in the majority (78%) of fractions the number of goals met by the adaptive plan is greater than that met by the scheduled plan. Fifteen percent of fractions have no difference in number of goals met by the adaptive and scheduled plans, and 7% have more goals met for the scheduled plan compared to the adaptive plan.

### 3.5 Frequency of adaptive plan selection

The frequency that the adaptive plan was selected for each treatment site is shown in Table 2. Overall the adapted plan was selected in 95% of fractions, with it being selected less frequently for prostate bed and node treatments.

**TABLE 2:** Percentage of fractions that the adaptive plan was selected for treatment

**Treatment site**	**Percentage that adapted plan was selected**
Intact prostate	98.8%
Intact prostate and nodes	98.7%
Prostate bed and nodes	89.4%
All sites	95.3%

### 3.6 Clinical goals per fraction

**FIGURE 3:**
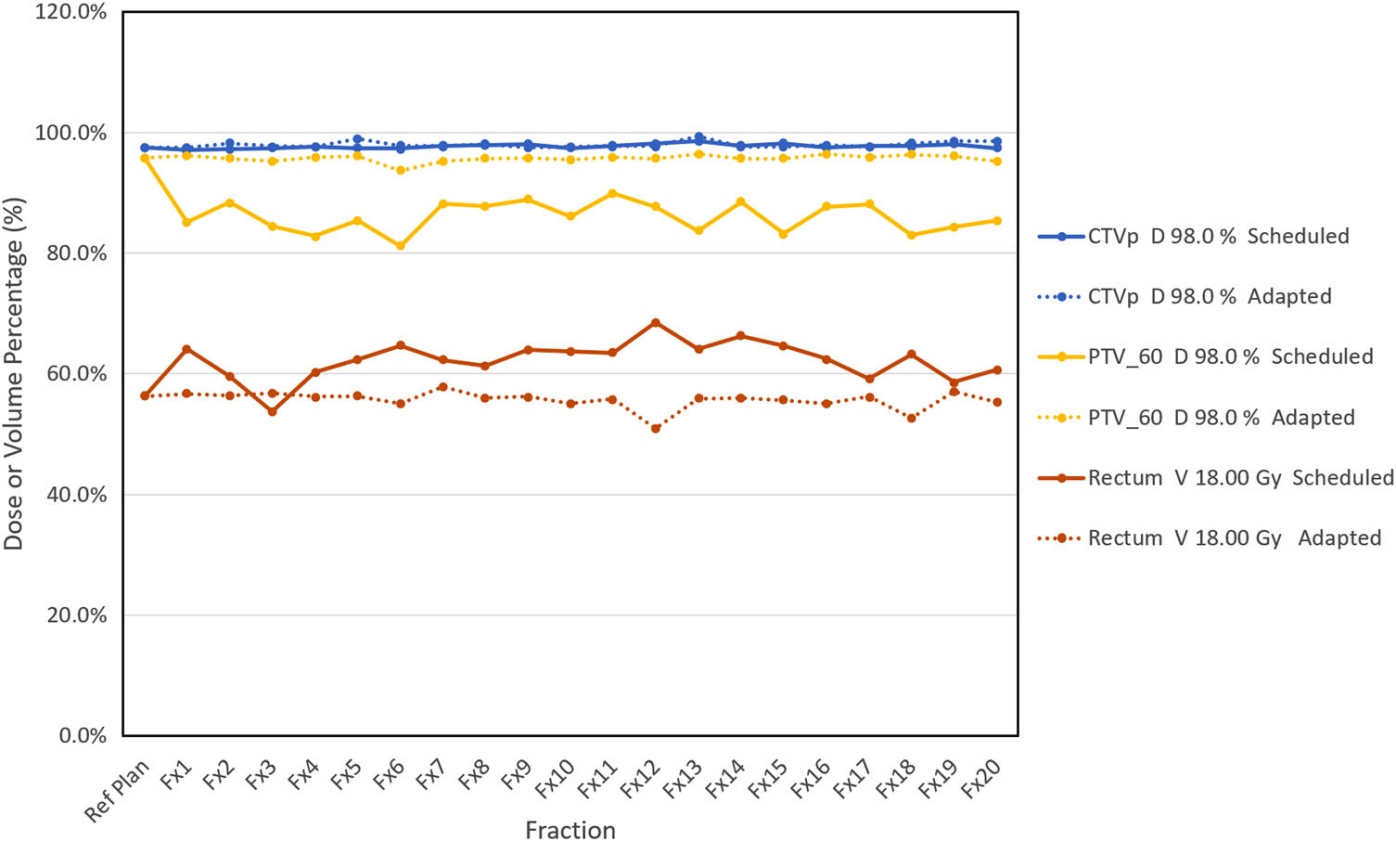
Selected plan parameters shown for each fraction for a representative prostate patient. Note the reference plan values (far left) match the values achieved by the adaptive plan more closely than the scheduled plan. CTVp and PTV_60 coverage is generally higher and rectum dose is generally lower for the adaptive plan for each fraction

Figure 2 shows that, for the majority of fractions, the adaptive plan meets a greater number of clinical goals than the scheduled plan. However, it does not display how the goals themselves change for a given case, or over the course of the treatment. Figure 3 shows a graph of selected CTV, PTV, and OAR clinical goals over each fraction of a treatment course for a representative clinical prostate patient. As can be seen in Figure 3, there is no long‐term trend in the goals over the treatment course, rather they vary day‐to‐day primarily due to bladder and bowel filling differences.

For the same patient, Table 3 displays which goals differ significantly between the scheduled and the adaptive plan. For most of the goals, the adaptive plan was shown to be superior, and in most of those cases the null hypothesis was rejected.

**TABLE 3:** Plan parameters achieved for scheduled and adapted plans for a representative prostate patient

				Scheduled plan		Adapted plan			
Structure	Goal	Variation	Reference plan	Median (Hodges‐Lehmann)	95% CI	Median (Hodges‐Lehmann)	95% CI	Superior plan	Statistical significance
CTVp	*D* 98.0% ≥ 98.0% P: 1	*D* 98.0% ≥ 95.0%	97.5%	97.7%	(97.3%–98.2%)	97.9%	(97.6%–98.8%)	Adapted	Fail to reject *H*_0_
CTVp	*D*_mean_ ≥ 100.0% P: 3	*D*_mean_ ≥ 99.0%	101.2%	101.3%	(100.9%–101.8%)	101.5%	(101.3%–101.8%)	Adapted	Reject *H*_0_
CTV_SV Prox	*D* 98.0% ≥ 98.0% P: 1	*D* 98.0% ≥ 95.0%	99.4%	98.8%	(97.9%–99.5%)	99.5%	(98.5%–100.6%)	Adapted	Reject *H*_0_
CTV_SV Distal	*D* 98.0% ≥ 98.0% P: 1	*D* 98.0% ≥ 95.0%	118.9%	118.9%	(117.6%–119.6%)	117.5%	(115.6%–119.3%)	Scheduled	Reject *H*_0_
PTV_60	*D* 98.0% ≥ 95.0% P: 1	*D* 98.0% ≥ 90.0%	95.8%	86.1%	(82.8%–89%)	95.8%	(94.7%–96.3%)	Adapted	Reject *H*_0_
PTV_60	*D* 95.0% ≥ 98.0% P: 1	*D* 95.0% ≥ 95.0%	97.0%	91.9%	(89.6%–93.9%)	97.0%	(96.5%–97.6%)	Adapted	Reject *H*_0_
PTV_60	*D*_min_ 0.30 cm^3^ > 100.0% P: 3	*D*_min_ 0.30 cm^3^ ≥ 60.0%	89.9%	74.0%	(61.3%–78.4%)	87.8%	(84.7%–89.7%)	Adapted	Reject *H*_0_
PTV_60	*D* 1.0% ≤ 105.0% P: 4	*D* 1.0% ≤ 107.0%	103.0%	103.4%	(103%–104%)	103.1%	(102.9%–103.3%)	Adapted	Reject *H*_0_
PTV_60	*D*_max_ 0.30 cm^3^ ≤ 107.0% P: R	*D*_max_ 0.30 cm^3^ ≤ 110.0%	103.5%	104.1%	(103.6%–104.7%)	103.7%	(103.5%–103.9%)	Adapted	Reject *H*_0_
PTV_50	*D* 98.0% ≥ 95.0% P: 2	*D* 98.0% ≥ 90.0%	114.1%	102.9%	(99.3%–106.4%)	111.9%	(109.4%–113.7%)	Adapted	Reject *H*_0_
PTV_50	*D* 95.0% ≥ 100.0% P: 3	*D* 95.0% ≥ 95.0%	116.2%	109.9%	(107.3%–112.2%)	115.8%	(114.8%–116.5%)	Adapted	Reject *H*_0_
Rectum	*V* 57.00 Gy < 15.0% P: 2	*V* 57.00 Gy ≤ 16.0%	1.3%	0.6%	(0.1%–2.2%)	1.0%	(0.4%–2%)	Scheduled	Reject *H*_0_
Rectum	*V* 60.00 Gy < 3.0% P: 2	*V* 60.00 Gy ≤ 4.0%	0.0%	0.0%	(0%–0.4%)	0.0%	(0%–0%)	Adapted	
Rectum	*V* 28.00 Gy ≤ 58.0% P: 2	*V* 28.00 Gy ≤ 60.0%	31.9%	33.3%	(28.6%–37.3%)	32.6%	(29.1%–34.5%)	Adapted	Fail to reject *H*_0_
Rectum	*V* 18.00 Gy ≤ 60.0% P: 2	*V* 18.00 Gy ≤ 65.0%	56.3%	62.5%	(57.3%–66.3%)	55.9%	(53.1%–57.1%)	Adapted	Reject *H*_0_
Rectum	*V* 52.80 Gy < 30.0% P: 3	*V* 52.80 Gy ≤ 30.0%	4.3%	2.9%	(0.9%–5.1%)	4.4%	(2.3%–5.7%)	Scheduled	Reject *H*_0_
Rectum	*V* 48.60 Gy < 50.0% P: 3	*V* 48.60 Gy ≤ 50.0%	7.3%	5.3%	(2.3%–8.1%)	7.7%	(5.2%–9.4%)	Scheduled	Reject *H*_0_
Rectum	*V* 40.80 Gy < 55.0% P: 3	*V* 40.80 Gy ≤ 60.0%	13.0%	11.0%	(6.3%–14.3%)	13.7%	(10.2%–15.8%)	Scheduled	Reject *H*_0_
Bladder	*D* 0.30 cm^3^ ≤ 60.00 Gy (3.00 Gy/Fx) P: 1	*D* 0.30 cm^3^ ≤ 61.00 Gy (3.05 Gy/Fx)	2.94	2.96	(2.94–3)	2.94	(2.94–2.98)	Adapted	Reject *H*_0_
Bladder	*V* 60.00 Gy ≤ 5.0% P: 3	*V* 60.00 Gy ≤ 7.0%	0.0%	0.0%	(0%–0.1%)	0.0%	(0%–0.1%)	Adapted	
Bladder	*V* 48.60 Gy ≤ 25.0% P: 3	*V* 48.60 Gy ≤ 30.0%	12.6%	10.3%	(7.5%–13%)	12.6%	(8.4%–17.6%)	Scheduled	Reject *H*_0_
Bladder	*V* 40.80 Gy ≤ 50.0% P: 3	*V* 40.80 Gy ≤ 60.0%	18.7%	16.3%	(11.5%–21.5%)	17.9%	(11.8%–24.4%)	Scheduled	Reject *H*_0_
Penile bulb	*D* 0.30 cm3 < 60.00 Gy (3.00 Gy/Fx) P: 1	*D* 0.30 cm^3^ ≤ 61.00 Gy (3.05 Gy/Fx)	2.95	2.95	(2.93–2.98)	2.93	(2.91–2.94)	Adapted	Reject *H*_0_
Penile bulb	*D*_mean_ ≤ 48.00 Gy (2.40 Gy/Fx) P: 3	*D*_mean_ ≤ 52.50 Gy (2.63 Gy/Fx)	2.20	1.77	(1.49–1.99)	1.88	(1.55–2.22)	Scheduled	Fail to reject *H*_0_
Femur head right	*D* 0.30 cm^3^ < 50.00 Gy (2.50 Gy/Fx) P: 3	*D* 0.30 cm^3^ ≤ 52.00 Gy (2.60 Gy/Fx)	1.23	1.33	(1.28–1.37)	1.43	(1.32–1.52)	Scheduled	Reject *H*_0_
Femur head left	*D* 0.30 cm^3^ < 50.00 Gy (2.50 Gy/Fx) P: 3	*D* 0.30 cm^3^ ≤ 52.00 Gy (2.60 Gy/Fx)	1.65	1.56	(1.53–1.6)	1.50	(1.39–1.56)	Adapted	Reject *H*_0_
Sigmoid colon	*D* 0.30 cm^3^ < 59.00 Gy (2.95 Gy/Fx) P: 1	*D* 0.30 cm^3^ ≤ 60.00 Gy (3.00 Gy/Fx)	1.36	1.93	(1.5–2.33)	1.49	(1.25–2.12)	Adapted	Reject *H*_0_
Sigmoid colon	*V* 48.60 Gy < 50.0% P: 3	*V* 48.60 Gy ≤ 51.0%	0.0%	0.2%	(0%–0.9%)	0.0%	(0%–1.3%)	Adapted	Fail to reject *H*_0_
Sigmoid colon	*V* 40.80 Gy < 55.0% P: 3	*V* 40.80 Gy ≤ 60.0%	0.0%	1.0%	(0%–2.7%)	0.1%	(0%–3.4%)	Adapted	Reject *H*_0_

*Note*: Statistical significance is determined using a Wilcoxon‐signed rank test, the Hodges–Lehmann median is displayed, and Walsh averages were used to determine 95% confidence intervals.

### 3.7 Timing data

The timing data are shown in Table 4. The steps carried out during the emulator time included: AI contouring, adjustment of influencers, creation of targets and noninfluencer OARs and their subsequent review and editing, optimization of the adaptive plan, final dose calculation of scheduled and adaptive plan, and review and selection of the preferred plan for the treatment. This closely represents the time from initial CBCT acquisition to beam‐on, which is most relevant to intrafraction motion. The clinical treatment time recorded also included patient setup, patient‐specific QA, acquisition of verification imaging, and treatment. In a number of fractions, it also included time waiting for the oncologist to come to the treatment console, after the patient had been setup for the treatment.

**TABLE 4:** Timing data for retrospective and clinical patient fractions

**Treatment site**	**Retrospective data Adaptive time (average ± SD) (mm:ss)**	**Clinical data Adaptive time (average ± SD) (mm:ss)**
Intact prostate	15:21 ± 03:18	33:57 ± 05:13
Intact prostate and nodes	19:30 ± 04:06	34:12 ± 06:23
Prostate bed and nodes	21:20 ± 03:55	34:17 ± 07:23
All sites	19:11 ± 04:29	34:11 ± 06:34

## 4 DISCUSSION

The frequency of minor edits, or no edits, to influencer and target contours found in this study overall was 92% and 91%, respectively. This is broadly in agreement with that shown by Sibolt et al.^18^ who reported 76% of influencers requiring no or minor edits over a greater range of treatment types. The results of Sibolt et al. are also somewhat skewed by the inclusion of bladder cancer treatments with a catheter in place, which were noted to have performed poorly during AI contouring. The frequency of target edits is marginally higher than that seen in the study by Sibolt et al.^18^ for prostate (100% requiring no or minor changes), but that study did not include prostate bed cases, or separate nodal from primary CTVs in the data presented. The results show that the intact prostate CTVp required less edits than the prostate bed CTVp, indicating that the system performs more accurately where there is a GTV that is also an influencer, compared to the more variable prostate bed CTVs without a GTV. Even so, the reported frequency of editing of influencer and target contours was similar, and indicates an efficient workflow is possible. The previous study by Yoon et al.^19^ covered different anatomy, however the reported accuracy of contouring was broadly consistent with this study. The frequency of adaptive plan selection reported by Sibolt et al. was lower than found in this study (88% compared with 95% found here), although not inconsistent given the range seen for different treatment sites (89.4% for prostate and nodes).

The differences in contouring accuracy for the sigmoid colon seen in Figure 1d in prostate bed cases are thought at least partially to be due to more mobility in the sigmoid colon post‐prostatectomy. Due to the propagation of the sigmoid colon from the simulation CT to the CBCT using an elastic DIR (which is not affected by influencers) in an area of low HU contrast, the DIR poorly tracks sigmoid colon movement. Therefore, the daily sigmoid colon contour accuracy is highly dependent on the sigmoid position during the simulation CT, and specifically how representative it is of the average sigmoid colon position on the treatment. The data presented here include 125 fractions, but these correspond to just six simulation CT scans, and therefore are likely an insufficient sample size to predict frequency of sigmoid colon edits.

When edits are made to targets or noninfluencer OARs, the treatment time substantially lengthens, as the optimization process that is already underway during the contour review step is restarted. Therefore to maximize efficiency, ideally no edits are required, and even minor changes can be problematic if seen consistently. To avoid the frequent edits to the sigmoid colon, we propose to add a 5 mm sigmoid PRV to the workflow in future. This PRV structure would be used as a dose avoidance region and would not be regenerated daily based on the sigmoid colon, but rather propagated from the planning CT. This would allow the user to verify that the sigmoid is within the PRV daily, and thus not spend additional time editing the sigmoid colon which would force a re‐optimization of the plan. This alteration has the disadvantage that it is not adapting the plan to the daily position of the sigmoid colon but rather is avoiding dose in the whole region surrounding the sigmoid colon, and thus may unnecessarily compromise coverage. In future, an AI‐based sigmoid colon influencer would be a preferable solution.

The contouring results for the single patient with a hip prosthesis were poor and required extensive editing. While the beam arrangement used can be adjusted to avoid the prosthesis, the extended contouring time is likely to make the use of Ethos adaptive inadvisable for the treatment of patients with hip prostheses.

This study employed user‐reported frequency and magnitude of contour editing. It is acknowledged that this is an imprecise surrogate for automated contouring accuracy. A more rigorous method would be to use a quantitative analysis (e.g., Dice similarity coefficient) comparing the automatically generated contours without edits against contours generated by an expert user (or users). Apart from being prohibitively cumbersome for a large number of fractions in the current implementation of Ethos, this would only allow analysis of OARs (both influencers and noninfluencers), as the CTVs are generated based on the edited influencers. One of the reasons that edits were required for the CTVs less frequently than for influencers, is that errors in contouring had been fixed in the influencer step and those fixes had been propagated to the CTVs. This is the intended workflow and design of software, so to analyze CTV accuracy without influencer edits would overestimate the CTV errors that occur in the actual treatment workflow. The other major reason that no changes are required for CTVs more often than no changes for influencers is the different instructions given to users, specifically not to make changes to CTVs unless the change is clinically significant.

In a small number of fractions, it was noted that the scheduled plan had more goals met than the adapted plan. Further analysis of these cases indicated that this could be due to some of the goals becoming more contradictory in a particular fraction (e.g., a rectum maximum dose overlapping with a minimum dose to the PTV). Due to the hierarchical nature of the IOE used in Ethos, this can mean that goals below this contradiction are not fully optimized, and lead to the scheduled plan meeting more clinical goals than the adaptive plan.

The largest plan quality differences noted for any structure was for the PTV, which consistently showed significant improvements in the adaptive plan, also seen in other studies.^26^ This is anticipated, as the PTV is just a tool to ensure that the CTV is covered, and under a normal image‐guided treatment it is not expected that the PTV would be fully covered on any given fraction, so long as the CTV is covered. Differences in CTV coverage were much smaller between scheduled and adapted plans, which is indicative that the margins were suitable for nonadaptive treatments. This is expected as the margins were those previously used for IGRT and chosen to cover the CTV with expected setup uncertainties. Re‐evaluation of margins will be the subject of a future study and may lead to greater dosimetric improvements being observed. The large differences in plan quality reported in previous studies such as Ahunbay et al.^1^ were primarily achieved through margin reductions, and therefore were not seen here.

Other plan differences were more mixed, with the adaptive plan sometimes inferior to the scheduled plan for a given clinical goal. When this occurred, it was generally noted that the adaptive plan had already met the ideal planning goal. Note that with the exception of the PTV discussed above, Table 3 does not suggest that the statistically significant differences seen for the case shown will be replicated for all prostate adaptive cases. Rather, that the goals and priorities selected for this specific case led to statistically significant improvements and deteriorations in the areas shown. A different set of priorities and goals would likely lead to different statistically significant differences for other cases. Our study suggests that organs that change significantly day‐to‐day (such that a goal is exceeded) are likely to see the largest improvement with adaptive replanning. Additionally, the statistically significant results shown in Table 3 do not necessarily indicate that the differences are also clinically significant.

The fraction timing data are consistent with what has been reported by other studies.^18^, ^19^ As would be expected, sites with more structures and more frequent contour editing took longer. It is expected the fraction time will reduce as staff continue to gain experience with the system.

## 5 CONCLUSION

Significant improvements in dosimetry are possible using the adapted plan, when compared to the scheduled plan, with the Ethos OART system for intact and prostate bed radiotherapy. No change or minor edits were achieved in 92% of influencer contours and 91% of target contours. The adaptive plan was selected in 95% of fractions. The early data presented here will assist other users of the Ethos system in implementing online adaptive radiotherapy to the prostate.

## AUTHOR CONTRIBUTIONS

Mikel Byrne, Ben Archibald‐Heeren, Amy Teh, and Rhea Beserminji designed the study. Mikel Byrne, Ben Archibald‐Heeren, Yunfei Hu, Amy Teh, Rhea Beserminji, Emma Cai, Guilin Liu, and Angela Yates all participated in acquiring the data for the study, either by performing retrospective or clinical treatment fractions, or both. Data analysis was performed by Mikel Byrne, Ben Archibald‐Heeren, Yunfei Hu, James Rijken, Nick Collett, and Trent Aland. All contributing authors reviewed the manuscript and gave feedback on the findings.

## CONFLICT OF INTEREST

Icon group is a member of the Varian Adaptive Intelligence Consortium and has a partnership with Varian to provide radiotherapy equipment. Mikel Byrne, Ben Archibald‐Heeren and Amy Teh have also received honoraria for presenting on behalf of Varian Medical Systems.
